# Bovine Virus

**Published:** 1886-01

**Authors:** 


BOVINE VIRUS.

We call the attention of our readers to the new advertisement of Messrs. Wyeth & Bro., relative to their "Vaccine Farm." The majority of the profession have had long experience in the use of the preparations of this firm, and have thereby acquired a confidence in anything which bears their name.

We have ourselves used their vaccine virus with entire satisfaction, and, from the methods followed and preventives employed at their "model farm," we anticipate that the reports of failures, after the use of their virus, will be rare.

